# Network trade-offs and homeostasis in *Arabidopsis* shoot architectures

**DOI:** 10.1371/journal.pcbi.1007325

**Published:** 2019-09-11

**Authors:** Adam Conn, Arjun Chandrasekhar, Martin van Rongen, Ottoline Leyser, Joanne Chory, Saket Navlakha

**Affiliations:** 1 Integrative Biology Laboratory, Salk Institute for Biological Studies, La Jolla, California, United States of America; 2 Sainsbury Laboratory, University of Cambridge, Cambridge, United Kingdom; 3 Howard Hughes Medical Institute and Plant Biology Laboratory, Salk Institute for Biological Studies, La Jolla, California, United States of America; University of Oxford, UNITED KINGDOM

## Abstract

Understanding the optimization objectives that shape shoot architectures remains a critical problem in plant biology. Here, we performed 3D scanning of 152 *Arabidopsis* shoot architectures, including wildtype and 10 mutant strains, and we uncovered a design principle that describes how architectures make trade-offs between competing objectives. First, we used graph-theoretic analysis to show that *Arabidopsis* shoot architectures strike a Pareto optimal that can be captured as maximizing performance in transporting nutrients and minimizing costs in building the architecture. Second, we identify small sets of genes that can be mutated to shift the weight prioritizing one objective over the other. Third, we show that this prioritization weight feature is significantly less variable across replicates of the same genotype compared to other common plant traits (e.g., number of rosette leaves, total volume occupied). This suggests that this feature is a robust descriptor of a genotype, and that local variability in structure may be compensated for globally in a homeostatic manner. Overall, our work provides a framework to understand optimization trade-offs made by shoot architectures and provides evidence that these trade-offs can be modified genetically, which may aid plant breeding and selection efforts.

## Author summary

In both engineered and biological systems, there is often no single structure that performs optimally on all tasks. For example, a transport system that can very quickly shuttle people to and from work will often not be very cheap to build, and vice-versa. Thus, trade-offs are born, and it is natural to ask how well evolution has resolved trade-offs between competing tasks. Here, we use 3D laser scanning and network analysis to show that *Arabidopsis* plant architectures make Pareto optimal trade-offs, which means that improving upon one task requires a sacrifice in the other task. In other words, an architecture that performs better on both tasks cannot be built. We also identify a small set of genes that can change how the architecture prioritizes one task versus the other, which may allow for better crop design in the future. Finally, we show that two replicate architectures that look visually diverse (e.g., variation in size, number of leaves, number of branches, etc.) often prioritize each task similarly. This suggests that despite local variability in the architecture, there may be a homeostatic drive to maintain globally balanced trade-offs.

## Introduction

An essential challenge of plant science is to uncover quantitative principles that describe the shape of plant architectures, defined here as the three-dimensional aerial arrangement of branches, leaves, and flowers [1, 2]. Prior work has discovered several descriptors of plant form [3], including phyllotaxis [4, 5], fractal and self-similar branching [6–9], and allometric scaling of various plant traits, such as height, stem diameter, and leaf biomass [10–13]. Recently, we uncovered a principle describing how architectures trade-off between the amount of resources required to build an architecture versus how efficiently nutrients can be transported using that architecture [14].

A related challenge is to uncover the molecular mechanisms (genes) driving these forms and optimizations. There have been many instances where genes controlling architecture shape have been manipulated to increase yield, including in cereals [15], rice [16], sorghum [17], and maize [18–20]. More recently, genome-wide association studies (GWAS) have helped link genetic variants to complex traits in a variety of species [17, 18, 21], and *Arabidopsis* *thaliana* continues to be an important model for performing screening assays. The broad goal of these studies is to understand the genotype to phenotype map well enough that a specific trait can be bred in a species of interest.

Here, we attempt to bridge these two ends—finding optimization principles and their genetic basis—by studying a trade-off made by *Arabidopsis* shoot architectures. The architecture of a plant is used to acquire resources from the environment and to distribute nutrients amongst different organs [22, 23]. We view the architecture as a geometric graph (often called a “skeleton”), where nodes correspond to the 3D locations of the base of the plant, branch points, or leaves; and edges correspond to branches (stem, axillary inflorescences, petioles) connecting nodes. Using this representation, we study how the architecture balances between a performance-cost trade-off [14]. Optimizing performance here means minimizing the distance to transport nutrients (e.g., sugar, water, carbohydrates) from the leaves to the root system, and vice-versa, along the architecture. Reducing cost means minimizing the amount of resources required to build and maintain the architecture. These two objectives are in conflict with each other—an architecture with efficient nutrient transport may require significant resources to build, and vice-versa. In this work, we study how *Arabidopsis* plant architectures resolve this tension.

Overall, we offer the following contributions. First, we performed high-resolution 3D laser scanning of 152 *Arabidopsis* shoot architectures, including wildtype (Col-0) and 10 mutant strains, each with a small number of gene mutations. We demonstrate that all 152 architectures make *Pareto optimal* trade-offs between our measures of performance and cost. An architecture is Pareto optimal if it is impossible to find an alternative architecture that improves upon both objectives at once (i.e., improving one objective requires sacrifice in the other objective). We also show that achieving Pareto optimality is unlikely to occur by chance—suggesting that random evolutionary pressures would not likely generate Pareto optimal structures—but that there are nonetheless some natural instances where this principle is violated. Second, we identify mutants that have architectures where the weight prioritizing one objective over the other is significantly shifted compared to that of the wildtype architecture. This suggests that the corresponding genes exhibit some control over architectural form and may be helpful to breed or design plant structures optimized for different functions. Third, we show that this prioritization weight feature is highly consistent across replicates of the same genotype despite significant visual diversity. This suggests that within a genotype, local changes in plant structure are compensated for globally in a perhaps homeostatic manner to keep trade-offs consistent. We show that this weight feature may be a more reliable indicator of a genotype compared to other common plant phenotyping traits.

The trade-off studied here is only one of many possible trade-offs made by shoot architectures, and does not include other important plant functions, such as light capture or root-shoot allocation trade-offs. Nonetheless, the fact that plants robustly lie along a Pareto front defined by this trade-off raises new questions about the molecular and physiological bases of these trade-offs and how they may also encapsulate other biological functions.

## Results

### High-resolution 3D scanning of *Arabidopsis* shoot architectures

Here, we describe the experiments performed to capture and digitize 152 *Arabidopsis* shoot architectures. We scanned both wildtype strains and a set of 10 mutant strains, each with a few genes modified (Table 1, Methods). For the mutants, we focused on genes whose activity can affect branching and flowering of shoot architectures in *Arabidopsis* [24–27]. For example, the strigolactone genes (*max4* [28] and *d14* [29–31]) can modify axillary bud outgrowth. While these mutants are certainly not exhaustive, they are diverse, well-studied, and provide a reasonable benchmark to test the generality of an architecture design principle.

**Table 1:** Dataset statistics. The first column shows the name of the genotype and the number of replicates scanned in parenthesis. The number of rosettes refers to the number of rosette leaves. Volume is measured as the convex hull of the cloud points. Errors indicate standard deviation across replicates.

Genotype	Refs.	Pareto trade-off	Epsilon (*ϵ*)	Alpha (*α*)	Total length (mm)	Travel dist. (mm)	# of rosettes	Volume (cm^3^)
Col-0 (6)	—	1.374 ± 0.05	1.020 ± 0.01	0.143 ± 0.04	584.20 ± 203.17	2652.18 ± 1199.28	15.50 ± 2.43	16218.20 ± 10542.63
*brc1,2* (10)	[58, 59]	1.640 ± 0.13	1.023 ± 0.01	0.093 ± 0.02	715.03 ± 247.40	2529.58 ± 938.34	20.90 ± 3.62	10699.93 ± 7183.65
*bes1* (5)	[60]	1.476 ± 0.11	1.015 ± 0.01	0.138 ± 0.04	450.10 ± 38.40	2021.45 ± 298.95	13.40 ± 2.06	4649.74 ± 1620.87
*cry1* (9)	[61]	1.328 ± 0.08	1.015 ± 0.01	0.153 ± 0.05	502.94 ± 97.36	2160.00 ± 552.46	15.22 ± 1.75	11018.63 ± 4744.52
*cry1,2* (8)	[62]	2.250 ± 0.25	1.020 ± 0.01	0.045 ± 0.02	1857.37 ± 455.44	6192.03 ± 2171.58	51.00 ± 6.40	42514.46 ± 19917.37
*cry2* (9)	[62, 63]	1.845 ± 0.18	1.033 ± 0.02	0.070 ± 0.03	1634.31 ± 958.74	6774.20 ± 5096.34	38.22 ± 11.29	62662.07 ± 57339.15
*d14* (8)	[29, 57, 64]	1.721 ± 0.10	1.017 ± 0.01	0.095 ± 0.03	724.46 ± 305.90	3200.36 ± 1802.02	19.63 ± 2.23	6245.72 ± 3782.02
*d14pin3,4,7* (5)	—	1.381 ± 0.12	1.018 ± 0.01	0.170 ± 0.02	392.94 ± 134.30	1788.00 ± 989.57	15.20 ± 1.94	3496.69 ± 1786.43
*max4* (11)	[55, 65]	1.709 ± 0.12	1.014 ± 0.01	0.096 ± 0.02	627.75 ± 254.99	2736.45 ± 1749.55	21.09 ± 2.91	5140.44 ± 3096.55
*phyA* (8)	[55, 66]	1.590 ± 0.11	1.021 ± 0.02	0.113 ± 0.05	789.60 ± 314.37	3542.85 ± 2374.67	25.38 ± 2.23	17810.91 ± 14208.55
*pin3,4,7* (8)	[50, 67]	1.234 ± 0.10	1.056 ± 0.02	0.224 ± 0.06	400.75 ± 74.03	1938.01 ± 645.63	15.25 ± 1.79	6075.83 ± 2359.54

**Fig 1:**
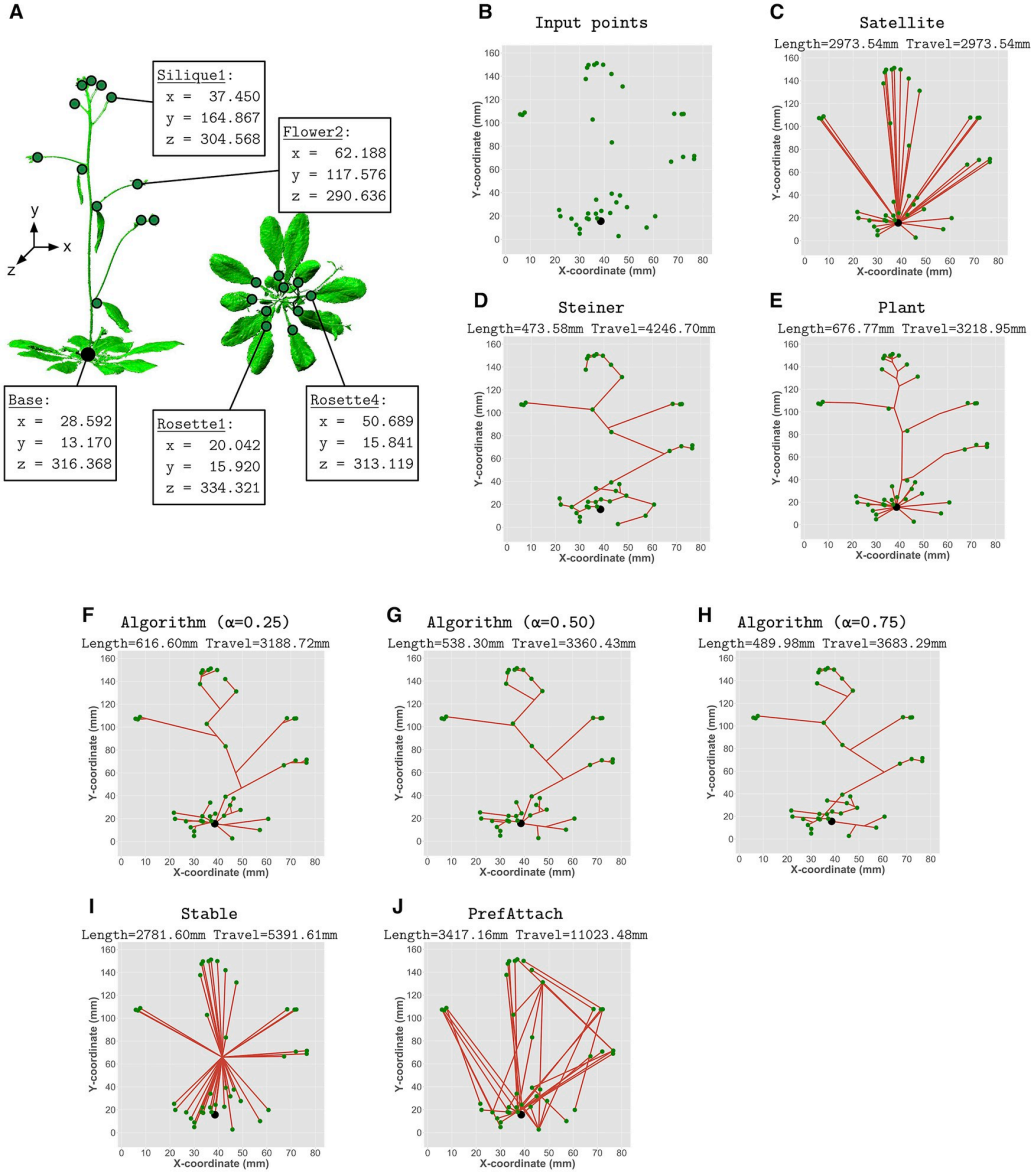
Graph-theoretic analysis of plant architectures. A) A 3D scan of a wildtype Columbia (Col-0) architecture with 219,792 cloud points. The (*x*, *y*, *z*) coordinates of the base of the plant and a few sample terminal points (rosette leaves, cauline leaves, siliques, flowers) are shown. B) Example input points from the 3D scan shown in panel A. The points shown represent the locations of the base of the stem (black point) and all terminal points (green points). C) The Satellite tree, which optimally minimizes travel distance. D) The Steiner tree, which optimally minimizes total length. E) The actual plant skeleton architecture connecting the input points through additional branch points. F–H) Example trees derived using the greedy algorithm for different values of the trade-off parameter, *α*. I–J) The Stable tree, and an example of a PrefAttach tree. The total length and travel distance of each architecture is shown in the title of each panel. For ease of visualization, panels B–J show 2-dimensional projections of the 3-dimensional trees. All graph-theoretic analysis is performed in 3-dimensions.

For each mutant, we performed 3D laser scanning of the shoot architecture (Fig 1A, Methods). We scanned at least five replicates of each strain (Table 1). Scans were performed after emergence of the inflorescence (Methods).

From each scan, we manually traced the skeleton architecture for the plant, including the curvature of branches. This tracing generated a graph *G* = (*V*, *E*), where the vertices *V* = (*v*_0_, *v*_1_, …, *v*_*n*_) represent the 3D locations of the base of the plant (*v*_0_) and the *n* terminal points (*v*_1_, *v*_2_, …, *v*_*n*_) correspond to the locations of all leaves, including rosettes leaves and cauline leaves (leaves on the inflorescence stem), flowers, or siliques (Fig 1A and 1B). Additional branch points may also be present in the skeleton, though these points are not provided as part of the input, *V*. The set of edges *E* correspond to the branches (stem, axillary inflorescences, petioles) connecting two nodes. Each edge has a length equal to the Euclidean distance between the two nodes it connects. Branches are considered in 1-D (length only). All edges are treated as undirected, as nutrients can flow in either direction.

### A computational framework for analyzing architecture trade-offs

Here, we describe a graph-theoretic method to test how well architectures balance between competing optimization objectives using the theory of Pareto optimality [32]. We previously described this framework [14] and elaborate it here briefly.

We study a performance-cost trade-off to measure how efficiently a plant’s architecture is used to distribute nutrients amongst organs [22, 23]. Performance (called *travel distance*) measures the distance that nutrients must travel from each terminal point (rosette leaf, cauline leaf, flower, or silique) to reach the base of the plant, or vice-versa, along the architecture. Biologically, this is related to measures of metabolic energy and hydraulic resistance required to transport sugars, nutrients, and water between the leaves and the root system [23, 33–37]; travel distance is also related to time delays in wound signaling responses, which can affect healing rates [38]. Cost (called *total length*) measures the total length or biomass of the skeleton. Biologically, this is related to the amount of resources (e.g., carbon) needed to build the architecture [39]; minimizing length can also aid in posture control by minimizing the amount of weight that needs to be supported [40, 41]. Thus, these two graph-theoretic measures relate to currencies known to affect biological function. However, by no means do they encapsulate all functions of plant architectures, nor do they directly capture all features of the plant phenotype space because, for example, the full range of travel paths to each terminal are not available due to the modular, meristem-driven nature of plant development. Instead, we accept that plants have evolved some mechanisms to determine how many leaves to generate and where each leaf should be placed. Given this, we study how optimal is the branching structure that is used to connect the given set of leaf points.

Mathematically, these two measures can be defined as:

$$\begin{array}{cc} \text{Travel}(G) & =\sum_{i=1}^{n}{\text{dist}}_{E}\left(v_{0},v_{i}\right) \end{array}$$ (1)

$$\begin{array}{cc} \text{Length}(G) & =\sum_{j}\left|E_{j}\right| \end{array}$$ (2)

The function dist_*E*_(*v*_0_, *v*_*i*_) computes the graph distance between the base of the plant (*v*_0_) and the *i*^th^ terminal point; i.e., the sum of the edge lengths along the path from *v*_0_ to *v*_*i*_. The length |*E*_*j*_| of edge *j* equals the Euclidean distance between the two endpoints of the edge.

What architectures minimize these two objectives individually? The architecture that minimizes travel distance alone is called the *Satellite* tree (Fig 1C). This tree contains a straight edge from the base of the plant to each terminal point. The architecture that minimizes cost alone is called the *Steiner* tree (Fig 1D). A Steiner tree is a tree that connects all the input points using the smallest total branch length. This tree can include branch points that are not provided as input (*V*) that can help reduce the length of the connecting architecture. In *Arabidopsis*, the rosette structure is Satellite-like, whereas the inflorescence typically is not.

These two architectures are in conflict with each other: the Satellite tree minimizes nutrient transport distances but has a large total length, whereas the Steiner tree minimizes total length but can have a large transport distance for some terminals.

#### Trade-offs and Pareto optimality

How well are trade-offs made between two competing objectives? Intuitively, an architecture makes a *Pareto optimal* trade-off between two objectives if improving along one objective necessitates a loss in the other objective [42]. Otherwise, both objectives can be improved at once, which means that the architecture “could have done better”. Prior work has made arguments suggesting that, given enough selection pressure and genetic diversity, evolution may push biological systems to be Pareto optimal [32], where the weight prioritizing each objective may depend on what provides a greater fitness advantage for a particular genotype in a particular environment [14].

To formalize this, we introduce a simple way to combine both objectives (travel distance and total length) that weighs the contribution of each objective individually. The goal is to find a graph G* that minimizes the joint objective:

$$\begin{array}{cc} G^{*} & =\underset{G}{\mathrm{argmin}}\;\alpha (\text{Travel}(G))+\left(1-\alpha \right)(\text{Length}(G))\\ & =\underset{G}{\mathrm{argmin}}\;\alpha (\sum_{i=1}^{n}{\text{dist}}_{E}\left(v_{0},v_{i}\right))+\left(1-\alpha \right)(\sum_{j}\left|E_{j}\right|) \end{array}$$ (3)

Here, the parameter *α* ∈ [0, 1] controls how much weight is placed on each objective. If *α* = 1, the optimal architecture is the Satellite tree; if *α* = 0, the optimal architecture is the Steiner tree.

To generate an architecture (tree) that nearly minimizes Eq (3) for any value of *α* ∈ [0, 1], we use a simple greedy algorithm [14]. Briefly, the algorithm initializes the tree with just the root vertex (*v*_0_) in the tree, and the rest of the vertices (*v*_1_, *v*_2_, …, *v*_*n*_) outside the tree. In each step of the algorithm, an edge is added that connects a vertex outside the tree to a vertex inside the tree that minimizes Eq (3). Along each edge added, *k* Steiner vertices (i.e., branch points) are added equidistant from each other; these vertices can be used as branch points to connect unconnected vertices in subsequent steps. In experiments here, we set *k* = 10. The algorithm terminates after *n* steps, when each vertex is added to the tree. While this method is clearly not optimal—minimizing Eq (3) is NP-hard—Conn et al. [14] provide evidence that this greedy algorithm generates very close to optimal trees and that it outperforms prior heuristics for this problem. Example architectures generated using different values of *α* are shown in Fig 1F–1H.

**Fig 2:**
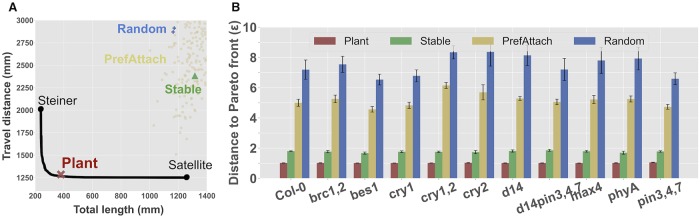
*Arabidopsis* shoot architectures are Pareto optimal. A) An example of the Pareto front (black curve) for an individual plant scan. The end-points of the Pareto front represent the individual optimals (Steiner and Satellite) of the two objectives. The red ‘X’ denotes the location of the plant. The green triangle shows the location of the Stable tree. The yellow circles show the locations of the Barabasi-Albert trees generated using the preferential attachment model. The blue diamonds show the locations of the Random tree. For the latter two methods, we generated 1000 trees; most of which lay outside the plotting area, implying that these architectures lie far away from the Pareto front. Overall, the plant architecture lies much closer to the Pareto front than other architectures. B) Summary of the entire dataset. The *x*-axis shows the list of all genotypes (Col-0 wildtype and 10 mutants). The *y*-axis shows the distance from the plant to the Pareto front; lower implies closer to the Pareto front, and thus, more optimal. Error bars indicate standard deviations over replicates. We compare the plant architecture versus Stable, PrefAttach, and Random architectures—each generating trees on the same set of input points. In all cases, the plant architecture lies significantly closer to the Pareto front than other architectures (*P* < 0.001).

By applying this algorithm to each value of *α* ∈ [0, 1], we can generate what is referred to as the *Pareto front*; i.e., the set of architectures for which improving along one objective requires a loss in the other objective. An example Pareto front is shown as the black curve in Fig 2A. If an architecture lies on the Pareto front, it means that there is no way to reconfigure the edges such that both objectives improve together. Evolutionarily, the idea is that architectures that do not lie on the Pareto front will be eliminated from the population over time.

Each plant scan has its own set of input points (*V*) specific to that scan. Thus, each plant scan has its own Pareto front, consisting of the trees generated for each value of *α* using *V* as input.

#### Analysis pipeline

From each individual plant scan, we extracted the 3D coordinates of the base of the plant (*v*_0_) and all the terminal points (*v*_1_, *v*_2_, …, *v*_*n*_). These *n* + 1 points were used as input to generate the Pareto front using the algorithm described above. The algorithm was run for 101 values of *α* ∈ [0, 1] in steps of 0.01. For each tree, we computed its total length and travel distance to generate a smooth Pareto front curve (e.g., Fig 2A). The Pareto front represents a set of trees, each of which approximately minimizes Eq (3) for a different value of *α*. To compare these trees with the plant, we traced the skeleton of the plant’s architecture that connects the same *n* + 1 points through any potential branch points, and then computed the plant’s total length and travel distance. The trees generated are all 3-dimensional but are displayed here in 2-dimensions for ease of visualization.

#### Computing the distance to the Pareto front

To determine how far away the plant architecture lay from the Pareto front, we first computed the total length and travel distance of each of the 101 Pareto optimal trees. Each of these trees has an (*x*, *y*) location on the Pareto front, where *x* is its total length and *y* is its travel distance. Thus, the Pareto front can be defined by a set of points, {(*x*_1_, *y*_1_), (*x*_2_, *y*_2_), …, (*x*_101_, *y*_101_)}. We similarly computed the total length and travel distance (*x*′, *y*′) of the actual plant architecture. Our goal was to determine how far away the point (*x*′, *y*′) was from the set of Pareto optimal points. We did this by scaling the Pareto front until it intersected with (*x*′, *y*′). That is, we multiplied each (*x*_*i*_, *y*_*i*_) point by a small scaling factor, *ϵ* > 1, until some point on the Pareto front intersected with (*x*′, *y*′). If the intersection occurred, for example, when *ϵ* = 2.0, then we say that the plant lies at a distance *ϵ* = 2 to the Pareto front, meaning that its total length and travel distance is collectively twice as bad as it could have been theoretically. The optimal value of *ϵ* is 1, meaning that the plant lies exactly on the Pareto front.

#### Comparing to baseline architectures

To assess whether the plant architecture lies significantly closer to the Pareto front than expected, we compared to three baseline architectures. The idea is to test how likely it is that trees generated according to other optimization criteria or models would be Pareto optimal.

The first baseline, called *Stable*, builds a structurally stable tree with even weight dispersion by computing the centroid of the *n* terminal points, adding a “stem” from *v*_0_ to the centroid, and then direct edges from the centroid to each of the terminal points (Fig 1I). The second, called *PrefAttach*, uses the Barabasi-Albert model [43] to generate a tree using a “rich-get-richer” mechanism with many potential hubs, which are commonly observed in efficient transport networks (Fig 1J). This algorithm starts with two random nodes connected by an edge. Then, in each iteration of the algorithm, it connects a new node to an existing node with probability proportional to the degree of the existing node. Parameters of the model were *n* = |*V*|, *m* = 1. The third baseline, called *Random*, creates a random spanning tree using Wilson’s loop-erased random walk algorithm [44]. All methods use the same points (*v*_0_, *v*_1_, …, *v*_*n*_) as input. For each baseline, we computed its travel distance and total length, and then the distance to the Pareto front using the method described above. For PrefAttach and Random, we average the distance over 1000 trees.

### *Arabidopsis* shoot architectures are Pareto optimal

Strikingly, all 152 *Arabidopsis* shoot architectures lay very close to the Pareto front, and much closer than the three baselines. For example, Fig 2A shows the analysis of an individual plant scan of a *d14* mutant that lies at a distance of *ϵ* = 1.016 to the Pareto front. On the other hand, the three other baselines lie much further away from the front: Stable (*ϵ* = 1.906), PrefAttach (*ϵ* = 4.027), and Random (*ϵ* = 6.037).

This observation—that plant architectures lie closer to the Pareto front than baselines—was consistent across all other mutant strains and for the wildtype strain (Fig 2B). Over all 152 architectures, the average distance to the Pareto front for the plant architectures was 1.020±0.016 compared to 1.742±0.139 for Stable, 3.990±0.555 for PrefAttach, and 6.089±1.087 for Random. Using a binomial distribution, where a success was equivalent to the baseline architecture having a smaller *ϵ* value than the plant, these differences are all significant (*P* < 0.001).

Critically, generating a Pareto optimal architecture is not trivial nor “inevitable”. Indeed, there are *n*^*n*−2^ possible spanning trees that can be formed given *n* vertices as input. For even small values of, say *n* = 20, that means there are 20^18^ = 2.62 × 10^23^ possible trees in phenotype space. The vast majority of these trees lie far from the Pareto front, as indicated by the Random baseline above. We also scanned two additional genotypes (*phot1-5phot2-1* and *phyB-9*) whose inflorescence often fell down because the plant was unable to support its own weight; interestingly, these plants also lay off the Pareto front (S1 Fig). This is in part driven by the fact that after falling down, the direction of light effectively changes and branches turn to a new direction. Thus, achieving Pareto optimality is not guaranteed for any branching structure, and it may be violated in some instances where the environment changes.

Our problem formulation considered hydraulic transport between the root and the shoot, but there are other forms of transport that may be important, such as sugar transport from leaves to flowers. The graph-theoretic structure that optimizes for minimizing sugar transport distances is a fully connected bi-partite graph from each leaf to each flower. This optimal, like the Satellite, would be costly to build. We compared the sugar transport distance and the construction cost of the bipartite graph versus that of the *Arabidopsis* architecture. We found that, over all mutants, the plant’s sugar transport distance was on average only 35% worse than optimal, but it was 5341% lower in cost. This observation does not strictly imply Pareto optimality between sugar transport performance and cost, but it does suggest that plants achieve a large “bang for the buck”; i.e., only losing 35% in performance but reducing cost by 5341%.

Overall, these results suggest that achieving well-balanced trade-offs may be an important growth principle for *Arabidopsis* shoot architectures. Further, these results suggest that the two objectives proposed here (travel distance, total length) may capture, or be correlated with, broad selective pressures that constrain architecture design.

### Identifying genes that can shift the trade-off balance

The analysis above showed that *Arabidopsis* architectures are nearly Pareto optimal, but where on the Pareto front do the architectures lie? This location may indicate niche specialization that varies based on the growth condition [14] or genetic background. This location also encodes a global feature of the architecture, indicating how this plant prioritizes each objective. Here, we explore how genetic modifications in our 10 mutants affect the weight prioritizing one objective versus the other. Overall, we find that mutations in even a single gene are sufficient to shift the architecture along the Pareto front one way or the other compared to the wildtype architecture.

To identify this prioritization weight, we introduce the *Pareto trade-off* feature for an architecture, *G*_Plant_:

$$\begin{array}{c} \text{Trade-off}\left(G_{\text{Plant}}\right)=\frac{\text{Length}\left(G_{\text{Plant}}\right)\;/\;\text{Length}\left(G_{\text{Steiner}}\right)}{\text{Travel}\left(G_{\text{Plant}}\right)\;/\;\text{Travel}\left(G_{\text{Satellite}}\right)} \end{array}$$ (4)

The numerator quantifies the excess length of the plant compared to the optimal minimum length of the Steiner tree. Similarly, the denominator quantifies the excess travel distance of the plant compared to the optimal minimum travel distance of the Satellite tree. A high value of this feature (i.e., a large numerator and small denominator) indicates that the plant prioritizes minimizing travel distance; a low trade-off value indicates the plant prioritizes minimizing total length. An alternative way to calculate the prioritization weight is to compute the value of *α* on the Pareto front that lies closest to the plant, though we found this feature was not as discriminative as the Pareto trade-off feature (Table 1, S2 Fig).

**Fig 3:**
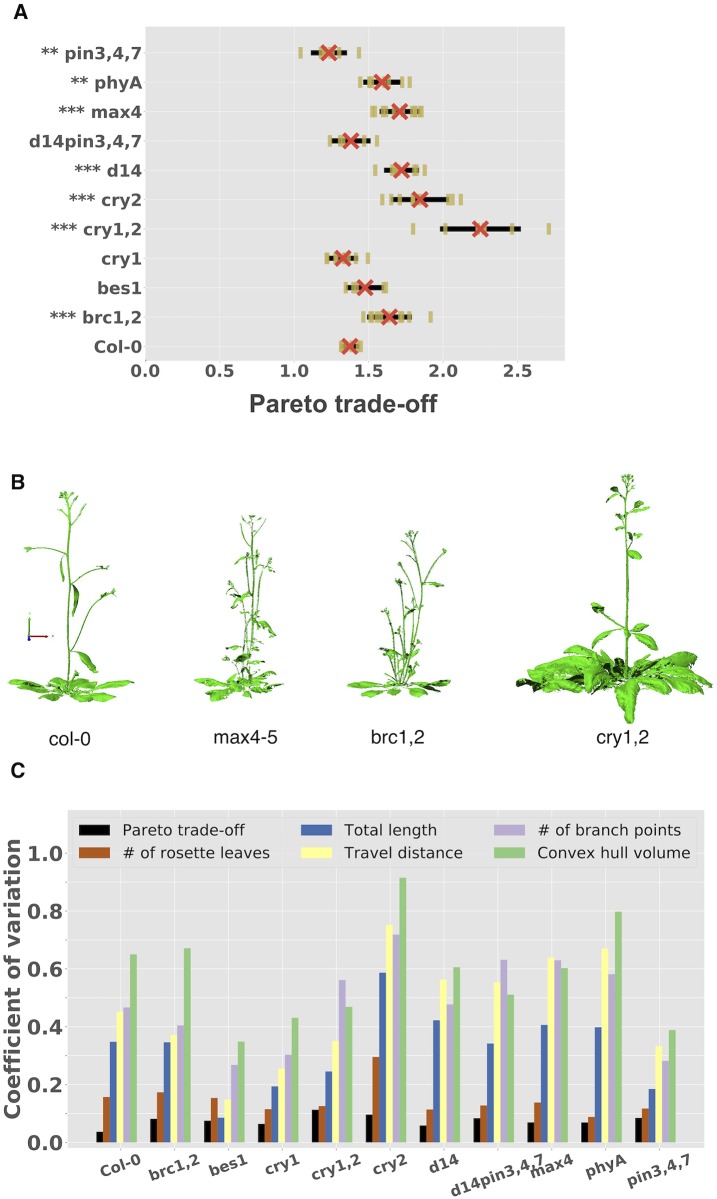
The Pareto trade-off feature better explains architecture diversity. A) The *y*-axis shows the genotype. The *x*-axis shows the standard deviation of the Pareto trade-off ratio for each genotype across at least five replicates per genotype. Each vertical dark yellow bar represents the trade-off of a single replicate. The red ‘X’ represents the mean over replicates. Black lines represent standard deviations. The mutants significantly different from wildtype are indicated by stars on the labels, with 2–3 stars, indicating a significance value of *P* < 0.01 and *P* < 0.001 respectively. B) Examples architectures for the Col-0, *max4*, *brc1,2*, and *cry1,2* mutants. C) The *x*-axis shows different mutants, and the *y*-axis shows the coefficient of variation. The Pareto trade-off feature has a consistently lower coefficient of variation compared to the other five plant features, indicating that it more robustly clusters a genotype compared to other traits.

We found that some mutants have significantly different trade-off values compared to the Columbia wildtype architecture (Fig 3A and 3B, Table 1). The trade-off ratio for the Col-0 wildtype was 1.374 ± 0.51. Mutants that shifted the architecture to the right of the curve (higher trade-off value) included *brc1,2*, *d14*, *cry2*, *cry1,2*, *max4*, and *phyA* (all *P* < 0.005, Kolmogorov-Smirnov test with Bonferroni correction using a threshold of 0.05). There was also one mutant that shifted the architecture left along the curve (*pin3,4,7*; *P* < 0.005).

Importantly, these results are not entirely driven by changes in the number of rosette leaves. We re-computed the Pareto trade-off feature for each mutant after excluding the rosette, focusing only on the inflorescence (S3 Fig). The *max4* and *d14* mutants still significantly shifted the architecture to the right, and the *pin3,4,7* mutant still significantly shifts the architecture left, along the Pareto front. Some mutants no longer show a significant difference from wildtype; e.g., the cry mutants, which are hallmarked by an increase in the number of rosette leaves at flowering [45]. Indeed, we found on average 38.22±11.29 rosette leaves for the *cry2* mutant, compared to 15.50±2.43 for the Col-0 wildtype (Table 1).

Overall, these results suggest that the modification of just a few genes (1–4) is sufficient to modify how architectures prioritize a global trade-off.

### The Pareto trade-off feature can be a robust descriptor of a genotype

Plant architectures can look highly diverse, even replicates with the same genetic background, grown in the same soil, in the same environment, and scanned at the same time [46]. Such visual diversity can be due to inherent stochasticity, noise, or other small changes in the local growth environment. This diversity can be problematic when trying to map a genotype-to-phenotype relationship because differences caused by genetic variables may be difficult to tease apart from those caused by other growth factors. Here, we asked whether the Pareto trade-off feature, which is a global feature of the architecture, is less variable across replicates of the same genotype compared to other common plant traits.

We quantified the variability of a feature using the *coefficient of variation* (CoV), which equals the standard deviation of the feature divided by its mean (over replicates). The CoV is a dimensionless parameter; lower CoVs are preferred and correspond to less variability in the feature.

We found that the Pareto trade-off feature had a lower coefficient of variation compared to five other traits: the total length, the travel distance, the number of rosette leaves, the number of branch points, and the convex hull volume occupied by the plant (Fig 3C). For example, for the Columbia wildtype architecture, the coefficients of variation were 0.037 (Pareto trade-off), 0.157 (number of rosette leaves), 0.348 (total length), 0.452 (travel distance), 0.467 (number of branch points), and 0.650 (convex hull volume). Similar trends were observed for each individual mutant (Fig 3C). The fact that total length and travel distance by themselves have such high CoV further highlights that what is consistent across these architectures is not these individual objectives, but rather their trade-offs.

We next tested how well the Pareto trade-off feature accounted for variation in the architecture of the same plant as it grew over time. In this case, the other features (such as the number of branch points) are expected to be variable because the plant is growing. The idea here is to test whether, as the shoot develops more biomass, does it still maintain a similar global trade-off ratio?

**Fig 4:**
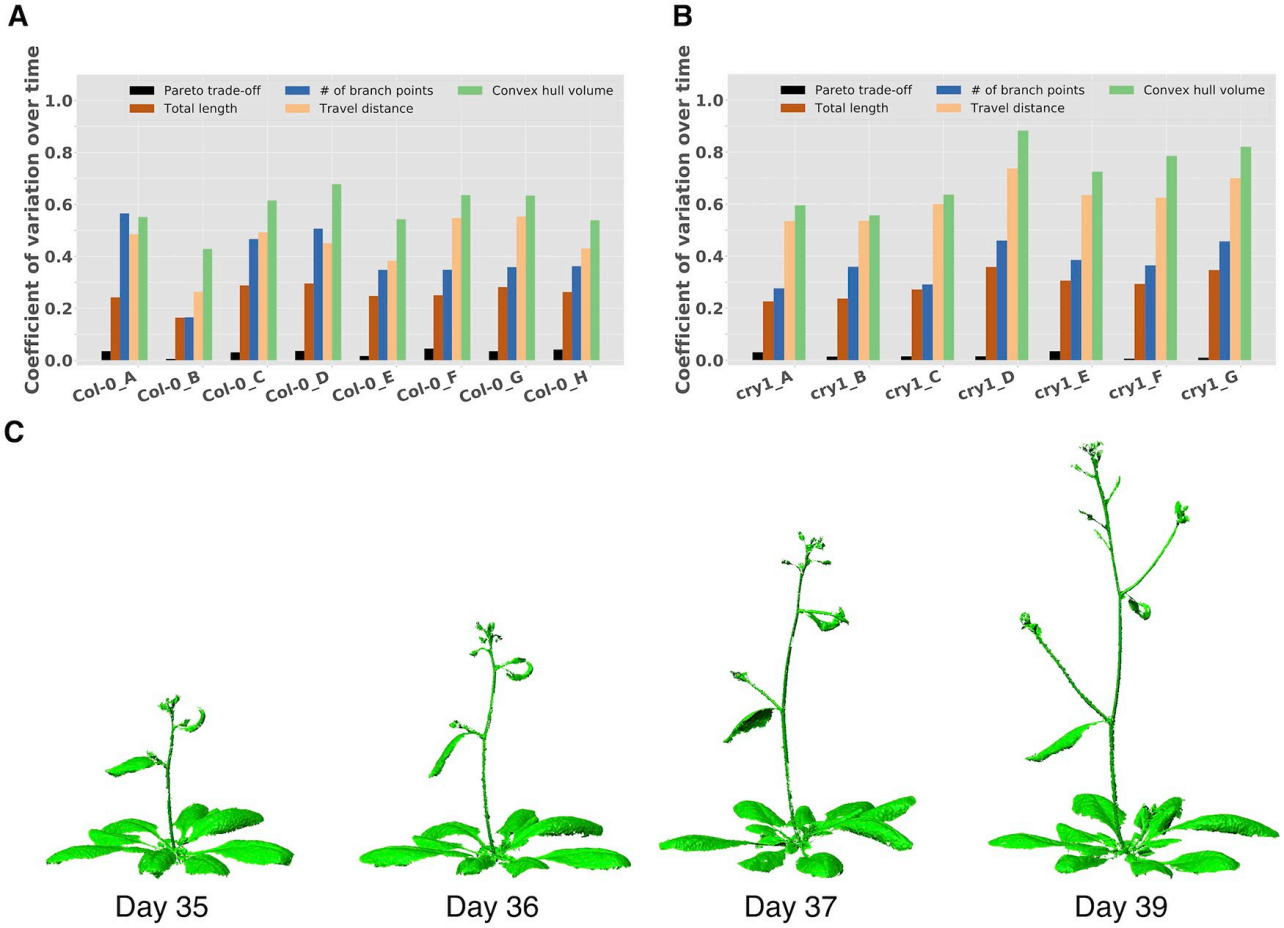
The Pareto trade-off is consistent through plant development. A) The *x*-axis shows the eight Columbia wildtype replicates. The *y*-axis shows the coefficient of variation across the time points for each replicate. B) Identical to panel A but using the *cry1* mutant. Throughout plant development, the Pareto trade-off remains much more consistent compared to other common traits. C) Four examples of time-series scans of Col-0_H.

To test this, we first scanned 8 individual Columbia wildtype plants over 3–5 time-points each, after the inflorescence emerged (Methods). For each scan, we computed the six features described above, and then we computed the coefficients of variation of these features over the 3–5 time points. As expected, the non-Pareto features demonstrate high variability as the plant grows; however, the Pareto trade-off feature was still highly consistent over time (Fig 4A and 4C). The coefficient of variation over time was 0.035 for Pareto trade-off versus 0.565 for the number of branches, 0.242 for total length, 0.485 for travel distance, and 0.551 for convex hull volume. We also repeated this time-series scanning experiment for 7 individual *cry1* mutant plants and found similar trends (Fig 4B).

The consistency of the Pareto trade-off feature, both across replicates and through portions of development between inflorescence emergence and the floral transition, suggest that despite growth-related changes, the trade-off between performance and cost remains relatively steady. In other words, local changes in the architecture, such as emergence and elongation of branches and the formation of new leaves, are compensated by a global change, in perhaps a homeostatic sense. This homeostasis is well-captured by the Pareto trade-off feature, and thus could help classify genotypes even if they look visually dissimilar.

## Discussion

We extended our description of plant architecture optimization as a network design problem [14] to the model species *Arabidopsis*. We tested if architectures of this species are also constrained to the set of design solutions that form the Pareto front. The Pareto front is a diverse landscape [47] of optimal solutions where selection pressure acts on two conflicting criteria; in our case, travel distance for nutrient transport and total length for construction costs. The Pareto front defines a set of possible architectures for which improving upon one function requires a sacrifice in the other, where the exact balance between these two functions can vary based on the environment or genotype.

We used graph-theoretic analysis to show that *Arabidopsis* shoot architectures fall along the Pareto front and that this is highly unlikely to occur by chance. By comparing the trade-offs employed by wildtype versus mutant *Arabidopsis*, we showed that the modification of a small number of genes (1–4) is sufficient to significantly shift how an architecture prioritizes one function versus the other. For example, for the *cry1,2*, *cry2*, *brc1,2*, *d14*, *phyA*, and *max4* mutants, the architecture is shifted to favor transport efficiency over total length; whereas for the *pin3,4,7* mutant, the opposite occurred. These genes may serve as candidates to breed specific features of interest in plants optimized for challenging environments. Our framework can also be used to screen for other genes that trigger branching related changes, coupled with high-throughput phenotyping techniques [48, 49].

We also showed that the Pareto trade-off feature was much less variable across replicates—i.e., the Pareto trade-off feature more tightly clustered architectures of the same genotype compared to other common plant traits—and it remained consistent through some portions of development of the same plant. This stability would not hold true through all of development; e.g., nearly all *Arabidopsis* begin as pure Satellites prior to the emergence of the inflorescence. However, we did observe stability of this feature through later shoot development (i.e., after the inflorescence emerged), suggesting that as leaves and branches emerged, maintaining a globally stable trade-off may be a growth constraint on the architecture as a whole. Overall, these results suggests that local stochasticity in common traits (e.g., the number of leaves or branches) may be compensated globally such that the trade-off between performance and cost is relatively stable. This highlights an example of the broader biological principle of “global stability despite local variability”.

Biologically, how do we interpret our finding that *Arabidopsis* shoot architectures are Pareto optimal? The two functions we studied (transport efficiency and construction costs) correlate with factors known to affect plant performance, and they represent common network design criteria used to evaluate other biological engineered branching structures [14]. However, these functions alone are likely insufficient to stratify the full diversity of shoot structures. For example, two plants with the exact same set of input points that are connected using different branch angles or branch curvature would generate the same Pareto front according to our model. However, branch angles can vary between mutants—e.g., the *pin3,4,7* mutant has wider branch angles and the *d14* mutant has shallower branch angles than the wild type [29, 50]—and such changes in branch angles can affect other architecture functions, such as light capture [51, 52]. Further, without including some pre-defined points to connect, then ideal plants that minimize total length and travel distance would be very small plants with leaves very close to the ground, which is clearly not realistic. Similarly, we couched transport efficiency in terms of the hydraulic conductance from the root to the shoot and vice-versa, but other forms of transport may also be important, such as sugar transport from leaves to flowers. More generally, two different (non-isomorphic) graphs can have the same travel distance and construction costs [53], suggesting that their differences may be related to other dimensions of optimization not considered here. While our simple one-parameter model captures significant variation observed across the 152 architectures studied here, additional optimization functions are possible to include within our framework by parameterizing them within Eq (3) and deriving a graph-theoretic algorithm to find Pareto optimal topologies for the corresponding higher order objective. We emphasize that our goal here was not to develop a sophisticated and highly-parameterized model of 3D shoot architectures that captures all of these features and functions. However, the fact that plants were Pareto optimal within this relatively simple abstraction suggests that the two functions we did study may be correlated with the ones we did not.

There are four directions for future work that can build off the framework developed here. First, the observation that *Arabidopsis* shoot architectures remain Pareto optimal over some periods of development raises an interesting computational problem that plants appear to solve: given an architecture at time *t*, how do you grow or extend the architecture to connect new terminal points such that the architecture remains Pareto optimal at time *t* + 1, without pruning or eliminating existing parts of the architecture? This problem is reminiscent of a classic computer science problem, called the online Steiner tree problem [54], but with an additional objective (travel distance). Relatedly, we assumed that the 3D locations of the leaves were provided as input, and we studied how branches connected these leaves. It would also be interesting to determine if the 3D location of the next leaf can be predicted [5] given that the architecture, after connecting the leaf, should be Pareto optimal. To address this problem, other functions would need to be taken into account, such as the ability for the leaf to intercept light. Second, we studied genetic variants with changes to a small number of genes; a more comprehensive effort could study hundreds of natural *Arabidopsis* variants [55] with more elaborate genetic changes. Such analyses could then use genome-wide association methods using the Pareto trade-off as a target feature to link genotype to phenotype to environment. Relatedly, we focused on *Arabidopsis* here because of the vast mutant library available; future studies could study similar trade-offs in other species to test the generality of our observations. Third, our graph-theoretic analysis only considered lengths (skeletons), ignoring the radii of branching elements. While skeleton-based analyses are common, including radii as weighted edges in our framework may further stratify architectures, for example, in instances of hypocotyl thickening [56]. Fourth, none of the mutants we studied pushed the architecture far off the Pareto front, unless the architecture fell down. This suggests that the regulatory networks responsible for Pareto optimal pattern formation may be governed by a larger set of interacting genes, which remain elusive.

## Methods

### Plant growth experiments

Experiments were performed using 10 mutants, each within the Columbia wildtype background (Table 1). S1 Table shows the full mutant name with alleles specified. All mutants are published, with the exception of *d14pin3,4,7*. This mutant was created by crossing *d14-1* [57] with the *pin3-3pin4-3pin7En* triple mutants [50]. Plants displaying combined phenotypes of *d14* and *pin3,4,7* were selected in the F2. Homozygous quadruple mutants were verified in F3 plants, using previously published genotyping strategies [50, 57].

Seeds were stratified in 1 ml of 0.1% agar and stored at 4C in the dark for 48 hours prior to planting. Seeds were transferred to 12-celled planting trays using a pipette into SunGro Propogation mix soil. The soil was moistened with water containing 0.12–0.24 oz/gallon fertilizer (Plantex, Canada), 0.12–0.24 oz/gallon fungicide (Heritage, England) and sprinkled with Marathon and Bugs Be Gone to prevent infestation from small insects. Seeds were placed under a light source with lid on the tray, which was removed post germination. Upon germination, 6 cells of the tray were removed in staggered fashion to eliminate crowding affects. Soil was watered as needed. Temperature of growth rooms were held at 20C with long day cycles: 16h day and 8h night. Each shelf held two trays and contained 4 bulbs, 3 white light and 1 fluorescent.

Due to differing developmental trajectories, it was difficult to select a fixed day post-germination to compare or ‘align’ the scan of each strain. Instead, we scanned the plant after the inflorescence emerged, and (with the exception of the plants shown in S1 Fig) while the shoot was still sustaining an upright posture. While some variation in features could be introduced based on the timing of the scan, we justified this approach by scanning wildtype Col-0 and *cry1* mutants roughly every day after the inflorescence emerged; using these time-series scans, we found that the trade-offs achieved are relatively consistent as the architecture grows during this period (Fig 4).

For the time-series scanning experiments, the Columbia wildtype architectures were grown and scanned in two separate experiments, the first with replicates A–C, and the second with replicates D–H. Five scans were performed per replicate, from days 32–36 post-germination for A–C, and from days 35–39 post-germination for D–H. For *cry1*, the 7 replicates were all grown and scanned over time in one experiment, from days 29–36 post-germination (excluding day 34).

### 3D scanning and skeletonization of shoot architectures

A high resolution blue-laser scanner (Edge Scan Arm HD, Faro Inc.) was used to non-invasively generate a 3D point cloud representation of the plant surface architecture. The scanner resolution is on the micrometer scale with errors in ±25*um*. The average number of cloud points per mutant was 292,247 with a minimum of 64,918 and maximum of 1,144,740 points. Full technical details of the scanner and scanning procedure were previously described [9, 14].

To generate the skeleton, we used Polyworks to select points at the base of the plant, the base of each leaf (cauline leaves, rosette leaves, siliques, and flowers), and each branch point along the stem. Additionally, points were picked along the curvature of the stem to capture tortuosity if applicable. Tracing through these points generated a skeleton representation of the plant architecture, where each point was represented as a node, and nodes were connected by edges. Each skeleton is a tree, meaning that is has no cycles and there is exactly one path between any two nodes.

## Supporting information

S1 Table Mapping from short mutant names used in this paper to mutants with full allele names. (TIFF)

S1 Fig Plants that lie off the Pareto front. Three examples of architectures that fell down and that lay away from the Pareto front. A–B) Two examples of a *phot1,2* mutant. C) Example of a *phyB* mutant. The scaled distances to the Pareto front for these three plants are: 1.209, 1.165, and 1.109, respectively. These are significantly further away from the Pareto front compared to the results in the main text (1.020 ± 0.016, averaged over all 152 scans).
(TIFF)

S2 Fig Variation in other plant features. In each panel, the *y*-axis shows the genotype, and the *x*-axis shows the standard deviation in a feature for each genotype across replicates. Each dark yellow bar represents the feature value of a single replicate. The red ‘X’ represents the mean over replicates. Black lines represent standard deviations. The mutants significantly different than wildtype (Col-0) are indicated by stars on the labels, with 2–3 stars, indicating a significance value of *P* < 0.01 and *P* < 0.001 respectively. The features are: A) *α* value. B) The number of branch points. C) The convex hull volume of the cloud points. D) The number of rosette leaves. E) The total length of the architecture. F) The travel distance of the architecture. Overall, we find larger variation in these features (compared to the Pareto trade-off feature), and we find fewer genotypes that show a significant difference from wild-type.
(TIFF)

S3 Fig Pareto optimality analysis excluding the rosette. A) The *y*-axis shows the genotype. The *x*-axis shows the standard deviation in the Pareto trade-off ratio for each genotype. Each dark yellow bar represents the trade-off of a single replicate. The red ‘X’ represents the mean over replicates. Black lines represent standard deviations. The significant mutants are indicated by stars on the labels, with 2–3 stars, indicating a significance value of *P* < 0.01 and *P* < 0.001 respectively. B) The *x*-axis shows different mutants, and the *y*-axis shows the coefficient of variation for five plant features (excluding the rosette leaves). The Pareto trade-off feature still achieves a low coefficient of variation, indicating that variability (not driven by the number of rosette leaves) is also better captured by the Pareto trade-off feature.
(TIFF)
