# Supplementary figures and images for: Network trade-offs and homeostasis in Arabidopsis shoot architectures
Source: PLoS Comput Biol. 2019 Sep 11;15(9):e1007325. doi: 10.1371/journal.pcbi.1007325 (PMC6738579; doi:10.1371/journal.pcbi.1007325)

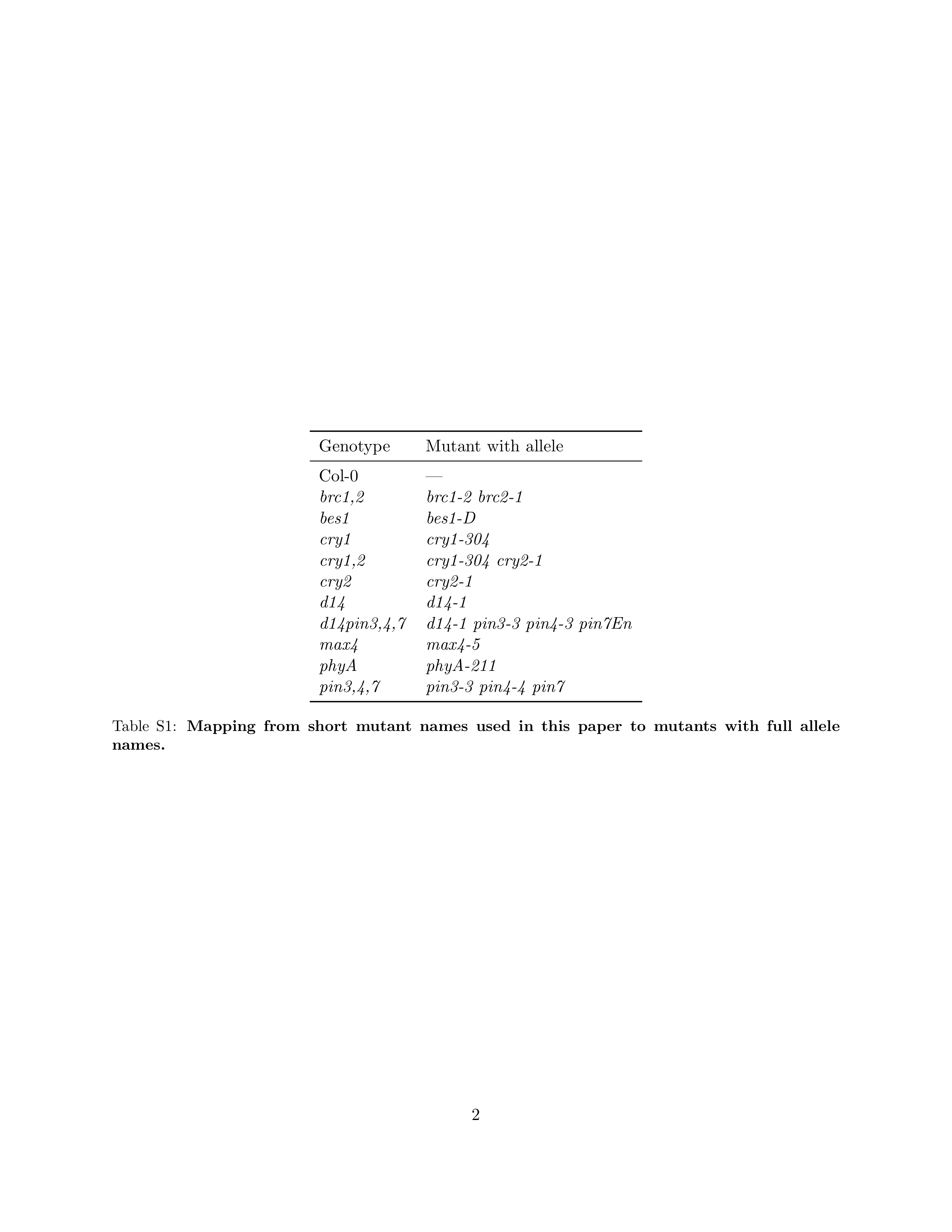

Supplement: S1 Table — (TIFF) [file pcbi.1007325.s001.tiff]

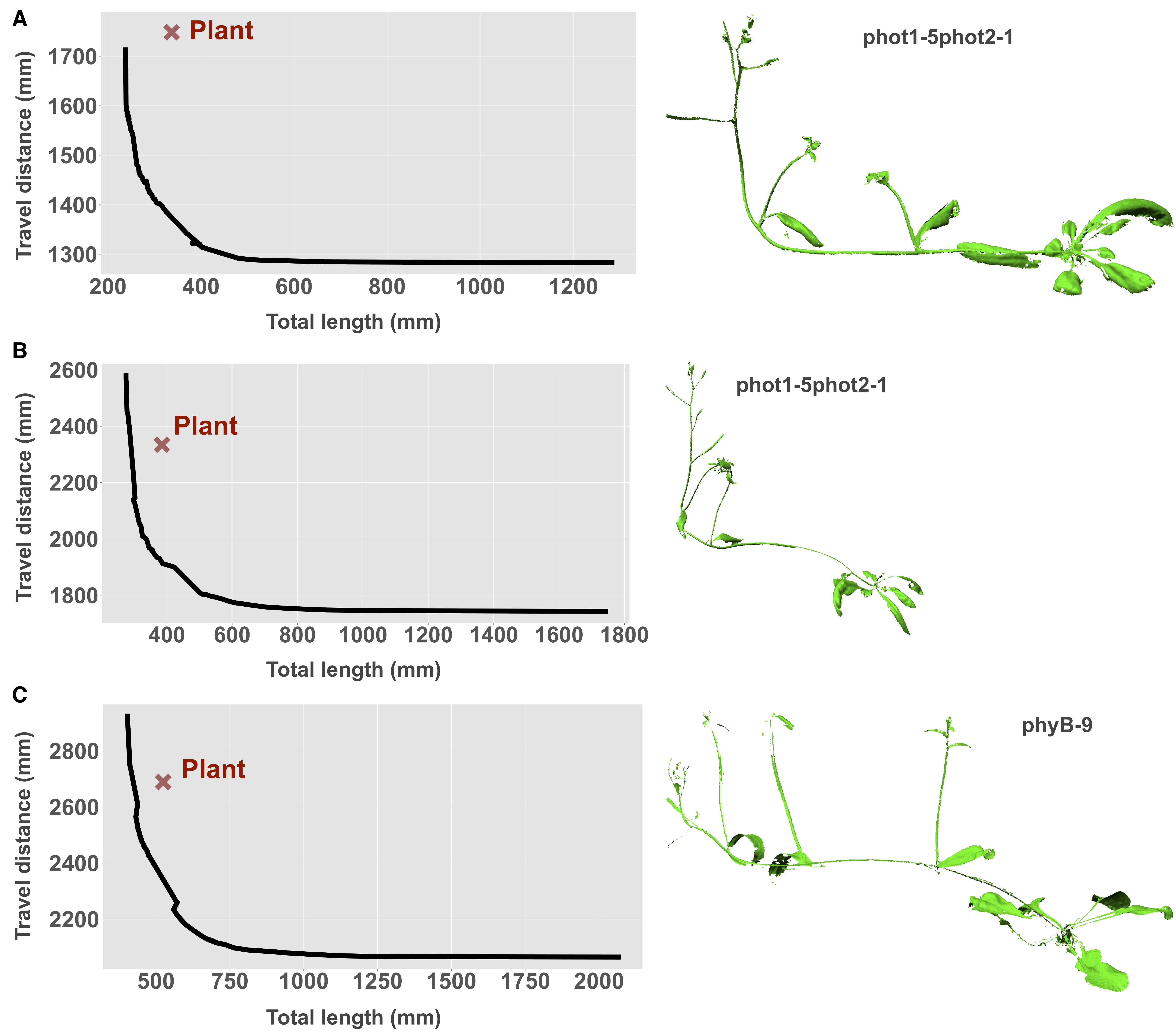

Supplement: S1 Fig — Three examples of architectures that fell down and that lay away from the Pareto front. A–B) Two examples of a phot1,2 mutant. C) Example of a phyB mutant. The scaled distances to the Pareto front for these three plants are: 1.209, 1.165, and 1.109, respectively. These are significantly further away from the Pareto front compared to the results in the main text (1.020 ± 0.016, averaged over all 152 scans). (TIFF) [file pcbi.1007325.s002.tiff]

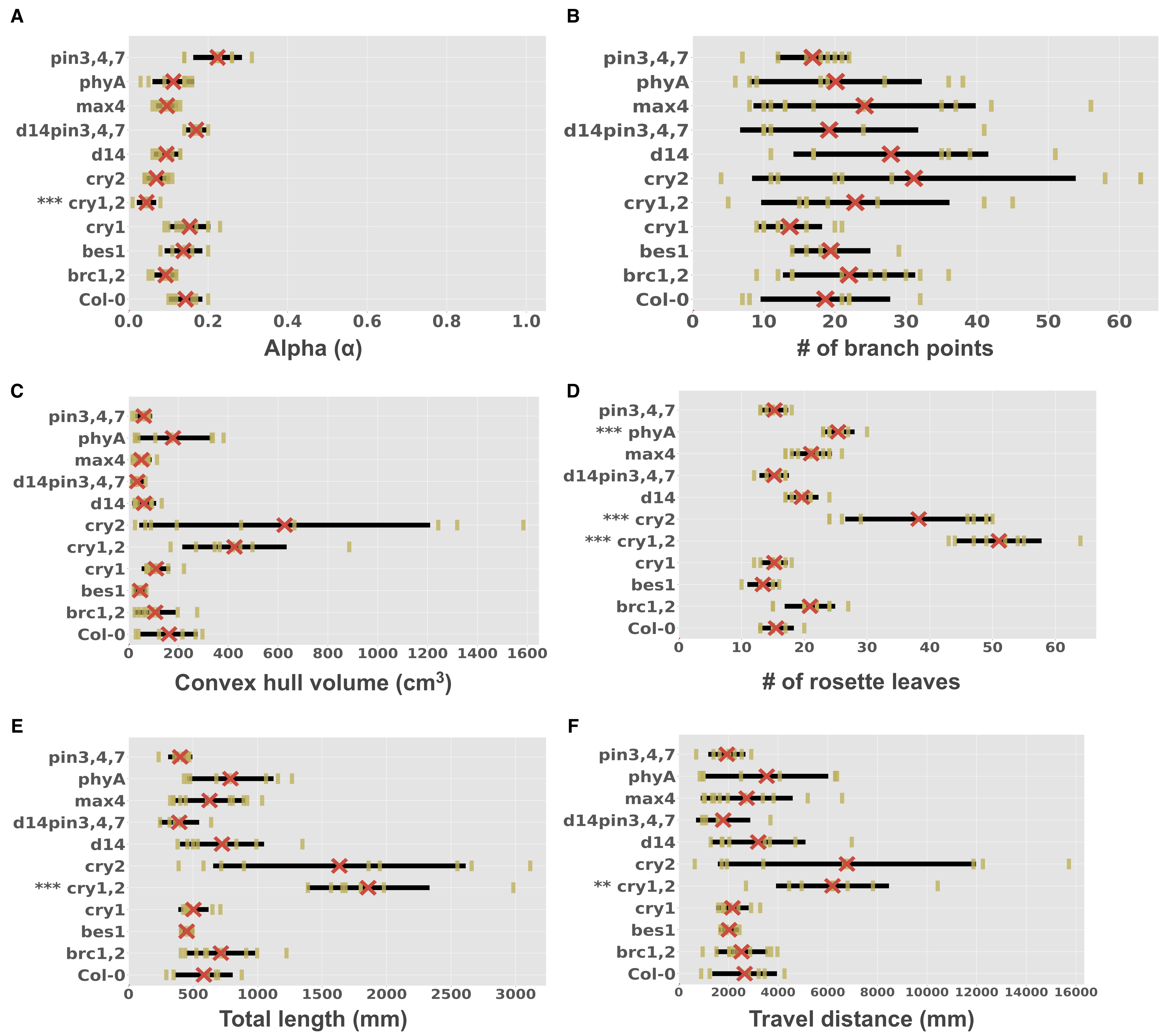

Supplement: S2 Fig — In each panel, the y-axis shows the genotype, and the x-axis shows the standard deviation in a feature for each genotype across replicates. Each dark yellow bar represents the feature value of a single replicate. The red ‘X’ represents the mean over replicates. Black lines represent standard deviations. The mutants significantly different than wildtype (Col-0) are indicated by stars on the labels, with 2–3 stars, indicating a significance value of P < 0.01 and P < 0.001 respectively. The features are: A) α value. B) The number of branch points. C) The convex hull volume of the cloud points. D) The number of rosette leaves. E) The total length of the architecture. F) The travel distance of the architecture. Overall, we find larger variation in these features (compared to the Pareto trade-off feature), and we find fewer genotypes that show a significant difference from wild-type. (TIFF) [file pcbi.1007325.s003.tiff]

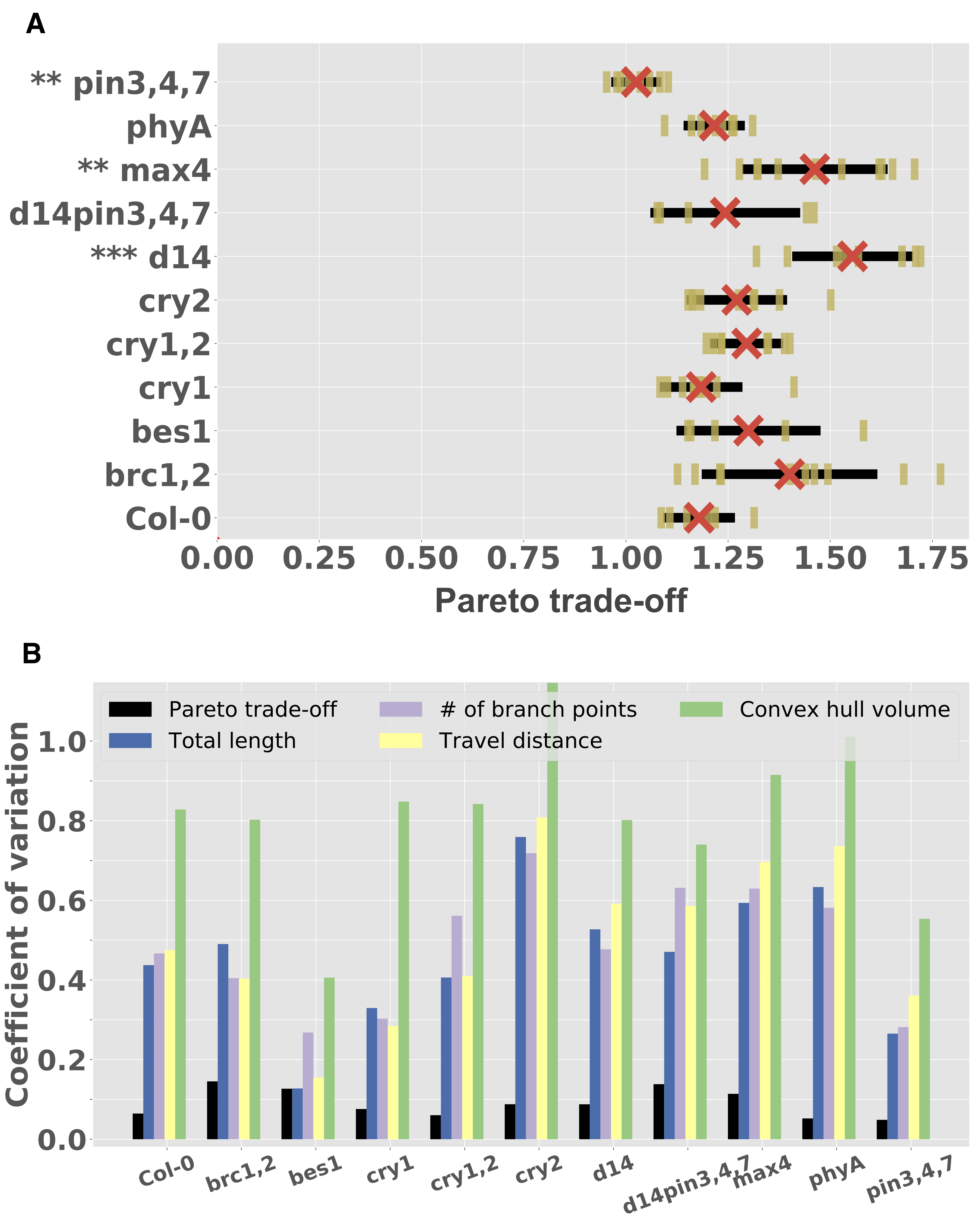

Supplement: S3 Fig — A) The y-axis shows the genotype. The x-axis shows the standard deviation in the Pareto trade-off ratio for each genotype. Each dark yellow bar represents the trade-off of a single replicate. The red ‘X’ represents the mean over replicates. Black lines represent standard deviations. The significant mutants are indicated by stars on the labels, with 2–3 stars, indicating a significance value of P < 0.01 and P < 0.001 respectively. B) The x-axis shows different mutants, and the y-axis shows the coefficient of variation for five plant features (excluding the rosette leaves). The Pareto trade-off feature still achieves a low coefficient of variation, indicating that variability (not driven by the number of rosette leaves) is also better captured by the Pareto trade-off feature. (TIFF) [file pcbi.1007325.s004.tiff]
